# The Association Between Serum Palmitic Acid and Thyroid Function

**DOI:** 10.3389/fendo.2022.860634

**Published:** 2022-05-03

**Authors:** Guowei Zhou, Yumeng Xu, Yuqing Zhai, Zhen Gong, Kun Xu, Gaoyuan Wang, Chenhua Sun, Chaoqun Ma

**Affiliations:** ^1^Department of General Surgery, Jiangsu Province Hospital of Chinese Medicine, Affiliated Hospital of Nanjing University of Chinese Medicine, Nanjing, China; ^2^Department of Colorectal Surgery, Jiangsu Province Hospital of Chinese Medicine, Affiliated Hospital of Nanjing University of Chinese Medicine, Nanjing, China

**Keywords:** palmitic acid, thyroid function, cross-sectional analysis, free thyroxine, NHANES

## Abstract

**Aim:**

Emerging evidence indicates that palmitic acid (PA) can regulate the progression and development of many diseases. However, the studies examining the association between PA and thyroid function remain sparse. We aimed to investigate the association between serum PA (sPA) and thyroid function in the US population.

**Methods:**

In this retrospective study, a cross-sectional analysis was performed using the data pooled from the database of the National Health and Nutrition Examination Survey (NHANES) from 2011 to 2012. The thyroid parameters investigated were mainly free triiodothyronine (FT3), free thyroxine (FT4), total T3 (TT3), TT4, thyroglobulin (Tg), thyroid-stimulating hormone (TSH), anti-thyroglobulin antibody (TgAb), and anti-thyroperoxidase antibody (TPOAb). The central sensitivity to thyroid function was evaluated by the thyroid feedback quantile-based index (TFQI), thyrotrophin thyroxine resistance index (TT4RI), and thyrotropin index (TSHI). The FT3 to FT4 ratio (FT3/FT4) was employed to evaluate peripheral sensitivity to thyroid hormones. Multiple imputation was applied to handle the missing data, and weighted multivariable linear regression, subgroup, and interaction analyses were then employed to estimate the association between sPA and thyroid parameters.

**Results:**

In the 737 adults, after adjusting covariates, we demonstrated a significant negative association between sPA and FT4 [β = -1.078, 95% confidence interval (CI): -1.729 to -0.427], as well as a positive relationship between sPA and FT3/FT4 ratio (β = 0.073, 95% CI: 0.044 to 0.102). These results did not change on multiple imputations. In the subgroup analyses, the associations were more significant in male and obese subjects.

**Conclusion:**

This investigation demonstrated the significant correlation between sPA and thyroid dysfunction, which suggested the close relationship between lipotoxicity and hypothyroidism or subclinical hypothyroidism. Future research is required to confirm these findings.

## Introduction

Palmitic acid (C16:0, PA) is the principal saturated fatty acid (SFA) of palm oil (PO) (1) and is also the most common plasma fatty acid (FA) in the human body (2, 3), representing 28%–32% of the total FAs in serum (4, 5). With increasing concerns about the relationship between nutrition and human health (6), there is growing evidence demonstrating that PA is much more than an energy source. The normal PA concentration is the basic guarantee for the multiple fundamental biological functions (3), whereas the overaccumulation of PA may result in adverse effects on human health (4). Emerging studies have linked elevated PA to multiple diseases (4), including cancers (7–10), diabetes (2, 11), metabolic syndrome (5, 12), cardiovascular diseases (CVDs) (13), autoimmune diseases (14), and neurodegenerative diseases (15, 16).

As is well known, thyroid hormones play a crucial role in regulating energy metabolism and cellular function (17). On the other hand, it is widely believed that lipotoxicity may lead to chronic cellular dysfunction and injury, with the thyroid gland considered to be the victim (18, 19). In a study by Araujo et al. (20), a high-fat diet could contribute to elevated serum thyroid-stimulating hormone (TSH) levels and increased systemic oxygen consumption. Shao et al. (21) reported that, in parallel with increased TSH levels, excess dietary high-fat lard intake could also induce decreased serum-free thyroxine (FT4) and total T4 (TT4) levels, as well as abnormal morphology in rats, which might be a long-term course. In addition, lipotoxicity was also correlated with the risk of subclinical hypothyroidism (SCH) (22). Furthermore, Zhao et al. (23) indicated that PA could downregulate the expressions and activity of the key molecules of thyroid hormone synthesis, including thyroglobulin (Tg), sodium iodide symporter (NIS), and thyroperoxidase (TPO). Moreover, lipotoxicity was also reported to be a significant risk factor in predicting extrathyroidal extension (ETE) of papillary thyroid microcarcinoma (PTMC) (24).

Although the excess intake of dietary fat is closely related to adverse health events, research investigating the association between serum PA (sPA) and thyroid function remains sparse. The current study aimed to examine the cross-sectional association between PA and thyroid parameters in a nationally representative sample of adults from the United States (US) using the National Health and Nutrition Examination Survey (NHANES) from 2011 to 2012.

## Materials and Methods

### Study Sample

NHANES is an ongoing cross-sectional survey conducted by the US Centers for Disease Control and Prevention, to assess the nutrition and health conditions of non-institutionalized citizens in the USA. The survey is performed biennially with a complex multistage cluster design, and the main sections of the database include demographics, dietary data, physical examinations, laboratory components, and multiple questionnaires. Before initiating interviews and data collection, the research protocol of NHANES has been approved by the Institutional Review Board (IRB) of the National Center for Health Statistics (NCHS), with written informed consent obtained.

**Figure 1:**
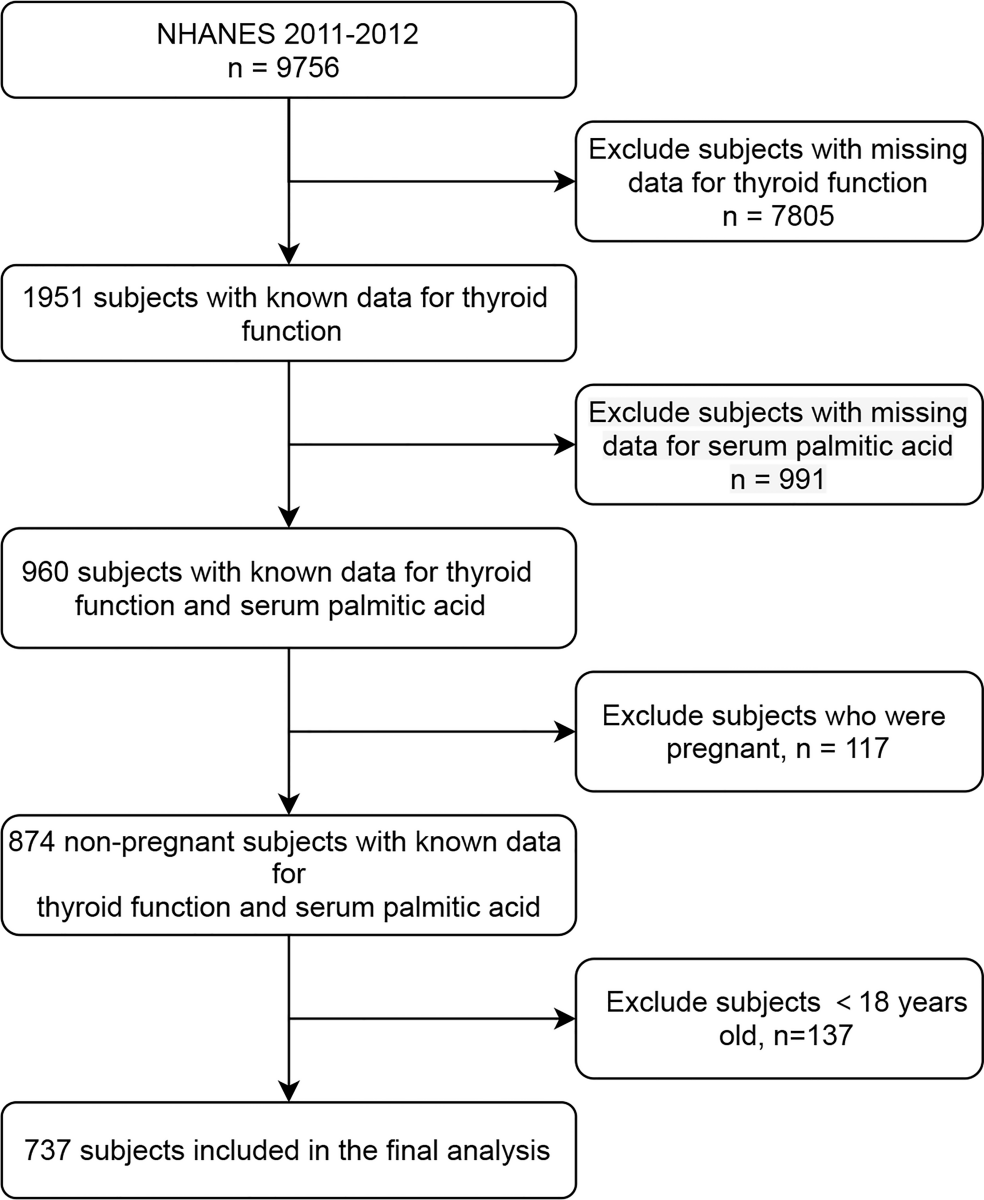
Study flowchart. NHANES, National Health and Nutrition Examination Survey.

The variables examined in the present study were pooled from NHANES 2011-2012 (‘‘G’’ data), since this cycle recorded full information on thyroid parameters and serum-free FAs. The participants who were not pregnant, aged 18 years or older, and had sPA, as well as thyroid function laboratory data, were included (**Figure 1**). The data were available on the NHANES website (https://www.cdc.gov/nchs/nhanes/).

### Measurement of Serum PA

The detailed method of measuring serum-free fatty acids, including serum PA, is described in previous articles (25, 26) and the Laboratory Method Files section of NHANES. In the current study, the natural logarithm (ln) transformation was applied to the sPA data (ranging from 1,220 to 13,200 µmol/l), which was then investigated as a continuous and categorical variable in tertiles (T1 = 7.11–7.76, T2 = 7.77–8.01, T3 = 8.02–9.49).

### Measurement of Thyroid Outcomes

The thyroid function parameters investigated in the present study included free triiodothyronine (FT3), FT4, TSH, total T3 (TT3), TT4, Tg, anti-thyroglobulin antibody (TgAb), and anti-thyroperoxidase antibody (TPOAb). The detailed specimen collection and processing instructions are available in the NHANES Laboratory/Medical Technologists Procedures Manual (LPM) and have been described in detail in the previous studies (27).

In the current work, the FT3/FT4 ratio was applied to reflect the converting activity of peripheral T4 to T3 (28). As for the central sensitivity to thyroid hormones, three indices were evaluated, including thyrotropin index (TSHI), thyrotrophin thyroxine resistance index (TT4RI), and thyroid feedback quantile-based index (TFQI). TSHI = ln TSH (mIU/L) + 0.1345 × FT4 (pmol/L) (29). TT4RI = FT4 (pmol/L) × TSH (mIU/L) (30). After transforming FT4 and TSH into quantiles between 0 and 1 by empirical cumulative distribution function (cdf), TFQI was then calculated according to the equation reported by Laclaustra et al. (31): TFQI = cdf FT4 (pmol/L) + cdf TSH (mIU/L)-1. Furthermore, thyroid autoimmunity was defined as TPOAb titers >9.0 IU/ml and/or TgAb titers >4.0 IU/ml (32). In addition, the urine iodine concentration (UIC) was employed to assess iodine conditions in participants, which was categorized into three groups: <99 µg/l, 99–199 µg/l, and >199 µg/l (33).

### Measurement of Covariates

The demographic values of age, gender, race/ethnicity, education, marital status, poverty-to-income ratio (PIR), medical history [including congestive heart failure, coronary heart disease, angina pectoris, heart attack, and cancer (25)], and tobacco and alcohol consumption were obtained from self-reported data using a standardized questionnaire. Education level was coded into three groups as less than high school, high school diploma, and more than high school, and PIR was categorized as 0–1 and >1 to assess the socioeconomic status (SES) of the participants. As proposed by NHANES, tobacco use was coded as never, former, and current status. In addition, alcohol use was assessed by asking the question “Had at least 12 alcohol drinks/1 year?”.

The physical examination information regarding mean arterial pressure (MAP) and body mass index (BMI) was acquired in the Mobile Examination Centers (MECs) by certified technicians, which were calculated by using the following formula: (1) MAP = [(diastolic blood pressure × 2) + systolic blood pressure]/3; (2) BMI = body weight (kg)/height^2^ (m^2^). In addition, BMI was coded into three groups: normal (BMI <25.0 kg/m²), overweight (25.0 kg/m²≤ BMI ≤29.9 kg/m²), and obese (BMI >29.9 kg/m²) (27).

Furthermore, the laboratory indices examined in the present study included aspartate aminotransferase (AST, U/L), alanine aminotransferase (ALT, U/L), creatinine (mg/dL), serum glucose (mg/dL), glycohemoglobin (%), and total cholesterol (TC, mg/dL), which were recorded by practiced technicians using standardized laboratory methods.

### Statistical Analysis

Since all of the continuous variables included in this work were abnormally distributed as evaluated by the Kolmogorov–Smirnov test, we applied the weighted Kruskal–Wallis (KW) test for the comparison of continuous variables to measure differences among ln sPA tertiles, which was presented as means and medians [interquartile ranges (IQRs)], while categorical variables were assessed by weighted chi-square test and were summarized using frequency counts and weighted percentages. Weighted multivariable linear and logistic regressions were then employed to investigate the association between ln sPA and thyroid function, including free and total T4 and T3, TSH, Tg, TgAb, TPOAb, thyroid autoimmunity, TFQI, TSHI, TT4RI, and FT3/FT4 ratio. Furthermore, to impute missing data, we employed multivariate multiple imputations with 5 replications and a chained equation approach (34). Interaction and stratified analyses were then conducted according to gender and BMI category. In addition, for addressing the non-linear relationship, smooth curve fittings and generalized additive models were employed. All data analyses were performed using the statistical software packages R 4.1.0 (http://www.R-project.org) and EmpowerStats (http://www.empowerstats.com, X&Y Solutions, Inc., Boston, MA), with a two-tailed *p* value <0.05 considered statistically significant.

## Results

### Baseline Characteristics of Participants

The study included 737 participants (range: 18–80 years) from NHANES 2011–2012, among which 380 were men and 357 were women. As demonstrated on **Table 1**, among sPA tertiles, the statistically significant (*p* < 0.05) thyroid parameters included FT4 and the FT3/FT4 ratio, which among all subjects were 10.80 (IQR: 9.80–11.90) pmol/l and 0.46 (IQR: 0.41–0.52), respectively. For covariates, the differences with statistical significance (*p* < 0.05) were age (*p* < 0.001), race/ethnicity (*p* < 0.001), smoking status (*p* = 0.009), alcohol use (*p* = 0.028), BMI (*p* < 0.001), waist circumference (*p* < 0.001), MAP (*p* < 0.001), AST (*p* < 0.001), ALT (*p* < 0.001), serum glucose (*p* < 0.001), glycohemoglobin (*p* < 0.001), and TC (*p* < 0.001). As shown in **Supplementary Table S1**, there were no statistical differences among covariates in 5 replications.

**Table 1:** Baseline characteristics of the NHANES (2011–2012) study population in ln sPA tertiles.

Characteristics	ln sPA tertiles					*p*-value
	Overall	T1		T2	T3	
n	737	238		253	246	
**Demographics**						
Gender (%)						0.625
Male	380 (51.56%) *^a^*	128 (53.78%)		125 (49.41%)	127 (51.63%)	
Female	357 (48.44%)	110 (46.22%)		128 (50.59%)	119 (48.37%)	
Age, years	46.00 [30.0–63.0] *^b^*	37.0 [24.0–59.0]		49.0 [33.0–64.0]	50.0 [36.0–63.0]	<0.001
Race (%)						<0.001
Non-Hispanic White	69 (9.36%)	24 (10.08%)		25 (9.88%)	20 (8.13%)	
Non-Hispanic Black	70 (9.50%)	17 (7.143%)		25 (9.88%)	28 (11.38%)	
Mexican American	302 (40.98%)	69 (28.99%)		112 (44.27%)	121 (49.19%)	
Other Hispanic	171 (23.20%)	74 (31.09%)		52 (20.55%)	45 (18.29%)	
Other races	125 (16.96%)	54 (22.69%)		39 (15.42%)	32(13.008%)	
Marital status (%)						0.403
Married	353 (50.50%)	108 (49.32%)		129 (53.98%)	116 (48.13%)	
Unmarried	346 (49.50%)	111 (50.68%)		110 (46.02%)	125 (51.87%)	
PIR (%)						
0–1	166 (24.67%)	54 (24.66%)		55 (24.12%)	57 (25.22%)	0.964
>1	507 (75.33%)	165 (75.34%)		173 (75.88%)	169 (74.78%)	
Education (%)						0.48
< High school diploma	172 (23.37%)	51 (21.52%)		60 (23.71%)	61 (24.79%)	
High school diploma	146 (19.84%)	44 (18.57%)		46 (18.18%)	56 (22.77%)	
> High school diploma	418 (56.79%)	142 (59.91%)		147 (58.11%)	129 (52.44%)	
Smoke (%)						0.009
Never	389 (55.57%)	135 (61.64%)		140 (58.58%)	114 (47.11%)	
Former	163 (23.28%)	50 (22.83%)		51 (21.34%)	62 (25.62%)	
Current	148 (21.15%)	34 (15.53%)		48 (20.08%)	66 (27.27%)	
Alcohol use (%)						0.028
<12 drinks per year	505 (74.82%)	141 (68.12%)		184(78.30%)	180 (77.25%)	
≥12 drinks per year	170 (25.18%)	66 (31.88%)		51(21.70%)	53 (22.75%)	
BMI, kg/m²	27.40 [23.70–32.10]	26.4 [22.40–30.00]		27.10 [23.80–31.80]	28.75 [25.00–33.78]	<0.001
WC, cm	96.50 [86.20–107.60]	91.90 [80.35–102.65]		97.00 [86.20–107.60]	100.60 [91.20–112.30]	<0.001
MAP, mmHg	86.00 [79.11–93.78]	84.22 [76.89–90.89]		86.22 [78.89–94.0]	88.00 [82.0–95.33]	<0.001
Congestive Heart failure (%)						0.756
No	19 (2.72%)	7(3.20%)		5 (2.10%)	7 (2.89%)	
Yes	680 (97.28%)	212 (96.80%)		233 (97.90%)	235 (97.11%)	
Coronary heart Disease (%)						0.999
No	29 (4.16%)	9 (4.11%)		10 (4.20%)	10 (4.17%)	
Yes	668 (95.84%)	210 (95.89%)		228 (95.80%)	230 (95.83%)	
Angina (%)						0.059
No	16 (2.28%)	3 (1.37%)		3 (1.26%)	10 (4.13%)	
Yes	684 (97.72%)	216 (98.63%)		236 (98.74%)	232 (95.87%)	
Heart attack (%)						0.169
No	29 (4.14%)	5 (2.28%)		10 (4.18%)	14 (5.79%)	
Yes	671 (95.86%)	214 (97.72%)		229 (95.82%)	228 (94.21%)	
Cancer (%)						0.723
No	53 (7.57%)	14 (6.39%)		19 (7.95%)	20 (8.26%)	
Yes	647 (92.43%)	205 (93.61%)		220 (92.05%)	222 (91.74%)	
**Laboratory indices**						
CR, mg/dL	0.84 [0.71–0.97]	0.84 [0.70–0.95]		0.83 [0.71–0.97]	0.84 [0.71–0.98]	0.902
Glucose, mg/dL	93.00 [86.00–10.00]	89.00 [82.25–99.75]		94.00 [87.0–102.0]	96.00 [88.25–107.00]	<0.001
Glycohemoglobin, %	5.50 [5.20–6.00]	5.40 [5.20–5.80]		5.60 [5.30–5.90]	5.60 [5.30–6.10]	<0.001
ALT, u/L	20.00 [16.00–28.00]	18.00 [15.00–23.75]		20.00 [16.00–25.00]	23.00 [17.00–34.00]	<0.001
AST, u/L	23.00 [19.00–27.00]	22.00 [18.00–26.00]		22.00 [19.00–26.00]	24.00 [20.00–30.00]	<0.001
UIC (%)						0.718
<99 ug/L	272 (37.31%)	89 (37.71%)		96 (38.55%)	87 (35.67%)	
99–199 ug/L	227 (31.14%)	68 (28.81%)		82 (32.93%)	77 (31.56%)	
>199 ug/L	230 (31.55%)	79 (33.48%)		71 (28.52%)	80 (32.77%)	
TC, mg/dL	189.00 [162.00–216.00]	158.50 [140.25–180.00]		192.00 [171.00–213.00]	217.00 [194.00–239.00]	<0.001
**Thyroid parameters**						
FT3, pg/mL	3.20 [2.96–3.46]	3.22 [3.01–3.49]	3.19 [2.95–3.43]		3.20 [2.95–3.46]	0.116
FT4, pmol/L	10.80 [9.80–11.90]	11.10 [10.10–12.08]	11.00 [9.80–11.90]		10.30 [9.30–11.58]	<0.001
TT3, ng/dL	114.00 [102.00–129.00]	118.00 [102.00–131.00]	113.00 [102.00–126.00]		116.00 [103.00–131.00]	0.190
TT4, ng/dL	7.97 [6.99–9.00]	7.99 [7.16–9.03]	7.89 [6.98–8.78]		8.06 [6.87–9.03]	0.478
TG, ng/mL	10.52 [5.98–17.78]	10.42 [6.10–15.80]	10.07 [5.40–18.08]		10.67 [6.57–18.46]	0.566
TGAb, IU/mL	0.60 [0.60–0.60]	0.60 [0.60–0.60]	0.60 [0.60–0.60]		0.60 [0.60–0.60]	0.114
TPOAb, IU/mL	0.50 [0.18–1.30]	0.50 [0.18–1.00]	0.50 [0.18–1.40]		0.60 [0.30–1.50]	0.183
TSH, µIU/mL	1.56 [1.13–2.33]	1.47 [1.10–2.26]	1.57 [1.10–2.32]		1.67 [1.18–2.45]	0.081
FT3/FT4	0.46 [0.41–0.52]	0.45 [0.41–0.50]	0.46 [0.40–0.51]		0.48 [0.43–0.54]	0.001
TSHI	1.92 [1.58–2.34]	1.87 [1.57–2.36]	1.94 [1.60–2.33]		1.93 [1.58–2.37]	0.903
TT4RI	16.85 [2.24–25.41]	16.24 [12.13–25.29]	17.12 [12.26–25.25]		17.38 [12.45–26.22]	0.665
TFQI	0.12 [-0.10-0.33]	0.14 [-0.10-0.35]	0.12 [-0.08-0.31]		0.09 [-0.15-0.32]	0.230
Thyroid autoimmunity (%)						0.150
No	637 (86.43%)	214 (89.92%)	216 (85.38%)		207 (84.15%)	
Yes	100 (13.57%)	24 (10.08%)	37 (14.63%)		39 (15.85%)	

a Actual frequency (weighted percentage).

b Weighted median [95% CI for median].

PIR, poverty-to-income ratio; BMI, body mass index; WC, waist circumference; MAP, mean arterial pressure; CR, creatinine; ALT, alanine aminotransferase; AST, aspartate aminotransferase; TC, total serum cholesterol; FT3, free triiodothyronine; FT4, free thyroxine; TSH, thyroid-stimulating hormone; TT3, Total T3; TT4, Total T4; TG, thyroglobulin; TgAb, anti-thyroglobulin antibody; TPOAb, anti-thyroperoxidase antibody, TFQI, the thyroid feedback quantile-based index; TSHI, thyrotropin index; TT4RI, thyrotroph thyroxine resistance index; UIC, urine iodin concentration.

### Association Between sPA and Thyroid Parameters

**Figure 2:**
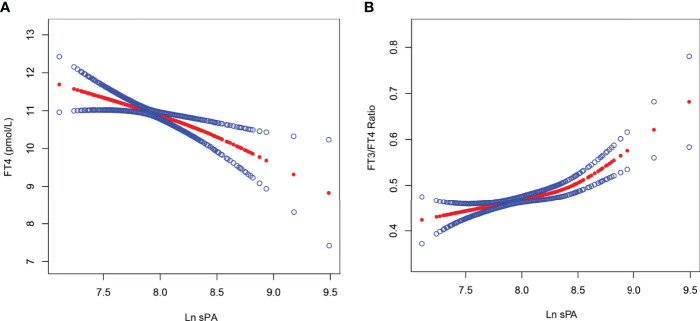
The association between ln sPA and FT4, as well as FT3/FT4 ratio. Solid red line represents the smooth curve fit between ln sPA and FT4 **(A)**, as well as FT3/FT4 ratio **(B)**. Blue bands represent the 95% of confidence interval from the fit. Age, gender, race/ethnicity, education, marital status, poverty-to-income ratio, mean arterial pressure, body mass index, waist circumference, alcohol use, smoke, alanine aminotransferase, aspartate aminotransferase, total cholesterol, glucose, glycohemoglobin, creatinine, and urine iodin concentration were adjusted. FT3, free triiodothyronine; FT4, free thyroxine; sPA, serum palmitic acid.

As demonstrated in **Table 2**, **Figure 2**, and **Supplementary Table S2**, sPA was significantly negatively and positively correlated with FT4 and the FT3/FT4 ratio, respectively, while the association between sPA and other thyroid parameters is listed in **Supplementary Table S3**. As shown in **Table 2**, the negative association between sPA and FT4 was present in the unadjusted model [β = -1.041, 95% confidence interval (CI): -1.465 to -0.617, *p* < 0.001], model 2 (β = -1.090, 95% CI: -1.516 to -0.663, *p* < 0.001), and model 3 (β = -1.078, 95% CI: -1.729 to -0.427, *p* = 0.001). Similarly, **Table 2** indicates a significant positive relationship between sPA and the FT3/FT4 ratio in the unadjusted model (β = 0.051, 95% CI: 0.032 to 0.071, *p* < 0.001), which existed after adjusting covariates, as indicated in model 2 (β = 0.064, 95% CI: 0.045 to 0.083, *p* < 0.001) and model 3 (β = 0.073, 95% CI: 0.044 to 0.102, *p* < 0.001). The significant relationship was also presented after categorizing ln sPA into tertiles (*p for trend* < 0.05), and the participants in the highest sPA tertile had a 0.667-pmol/l lower FT4 than those in the lowest sPA tertile (*p* = 0.009) in model 3, who also had a 0.036 greater FT3/FT4 ratio (*p* = 0.002).

**Table 2:** The association between ln sPA and FT4, FT3/FT4 ratio.

	Model 1 *^a^* β (95% CI) *p-value*	Model 2 *^b^* β (95% CI) *p-value*	Model 3 *^c^* β (95% CI) *p-value*
**FT4, pmol/L**			
ln sPA	-1.041 (-1.465, -0.617) <0.001	-1.090 (-1.516, -0.663) <0.001	-1.078 (-1.729, -0.427) 0.001
ln sPA categories			
Tertile 1	Reference	Reference	Reference
Tertile 2	-0.234 (-0.561, 0.093) 0.162	-0.218 (-0.544, 0.108) 0.190	-0.177 (-0.600, 0.246) 0.414
Tertile 3	-0.751 (-1.080, -0.422) <0.001	-0.726 (-1.057, -0.394) <0.001	-0.667 (-1.171, -0.162) 0.010
*p for trend*	<0.001	<0.001	0.008
**FT3/FT4 ratio**			
ln sPA	0.051 (0.032, 0.071) <0.001	0.064 (0.045, 0.083) <0.001	0.073 (0.044, 0.102) <0.001
ln sPA categories			
Tertile 1	Reference	Reference	Reference
Tertile 2	0.002 (-0.013, 0.017) 0.772	0.011 (-0.004, 0.025) 0.146	0.011 (-0.008, 0.030) 0.248
Tertile 3	0.030 (0.015, 0.045) <0.001	0.039 (0.024, 0.054) <0.001	0.036 (0.013, 0.059) 0.002
*p for trend*	<0.001	<0.001	0.001

a Model 1: no covariates were adjusted.

b Model 2: age, gender, and race/ethnicity were adjusted.

c Model 3: age, gender, race/ethnicity, education, marital status, poverty-to-income ratio, mean arterial pressure, body mass index, waist circumference, alcohol use, smoke, alanine aminotransferase, aspartate aminotransferase, total cholesterol, glucose, glycohemoglobin, creatinine, and urine iodin concentration were adjusted.

FT3, free triiodothyronine; FT4, free thyroxine; sPA, serum palmitic acid, CI, confidence interval.

Furthermore, the subgroup analysis indicated that gender was the interactive factor in the correlation between sPA and FT4 (*p for interaction* = 0.031), as well as the FT3/FT4 ratio (*p for interaction* = 0.036). As shown in **Table 3**, the association between sPA and FT4 was more significant in the male (β = -1.356, 95% CI: -2.203 to -0.509, *p* = 0.002, *p for trend* < 0.001) and obese (β = -1.160, 95% CI: -2.224 to -0.097, *p* = 0.034, *p for trend* = 0.018) participants in model 3; similar results were found in the relationship between sPA and the FT3/FT4 ratio, as the effect size was 0.082 for male (β = 0.082, 95% CI: 0.045 to 0.119, *p <*0.001, *p for trend <*0.001) and 0.071 for obese adults (β = 0.071, 95% CI: 0.024 to 0.117, *p* = 0.003, *p for trend* = 0.015).

**Table 3:** The association between ln sPA and FT4, FT3/FT4 ratio.

	FT4 (pmol/L)			FT3/FT4		
	Model 1 *^a^* β (95% CI) *p-value*	Model 2 *^b^* β (95% CI) *p-value*	Model 3 *^c^* β (95% CI) *p-value*	Model 1 *^a^* β (95% CI) *p-value*	Model 2 *^b^* β (95% CI) *p-value*	Model 3 *^c^* β (95% CI) *p-value*
**Subgroup analysis stratified by gender**						
**Gender = male**						
ln sPA	-1.458 (-1.989, -0.927) <0.001	-1.413 (-1.946, -0.881) <0.001	-1.356 (-2.203, -0.509) 0.002	0.074 (0.049, 0.099) <0.001	0.076 (0.053, 0.100) <0.001	0.082 (0.045, 0.119) <0.001
ln sPA categories						
Tertile 1	Reference	Reference	Reference	Reference	Reference	Reference
Tertile 2	-0.528 (-0.971, -0.084) 0.020	-0.507 (-0.950, -0.065) 0.025	-0.482 (-1.034, 0.070) 0.088	0.015 (-0.006, 0.036) 0.154	0.018 (-0.002, 0.038) 0.074	0.016 (-0.009, 0.040) 0.217
Tertile 3	-1.288 (-1.730, -0.847) <0.001	-1.197 (-1.641, -0.753) <0.001	-1.153 (-1.824, -0.481) <0.001	0.057 (0.036, 0.078) <0.001	0.057 (0.037, 0.076) <0.001	0.046 (0.016, 0.077) 0.003
*p for trend*	<0.001	<0.001	<0.001	<0.001	<0.001	<0.001
**Gender = female**						
ln sPA	-0.422 (-1.111, 0.267) 0.231	-0.615 (-1.329, 0.100) 0.092	-0.567 (-1.623, 0.489) 0.294	0.013 (-0.018, 0.044) 0.404	0.041 (0.009, 0.072) 0.011	0.054 (0.006, 0.103) 0.028
ln sPA categories						
Tertile 1	Reference	Reference	Reference	Reference	Reference	Reference
Tertile 2	0.124 (-0.352, 0.601) 0.609	0.021 (-0.465, 0.507) 0.933	0.068 (-0.610, 0.747) 0.843	-0.011 (-0.033, 0.010) 0.294	0.002 (-0.019, 0.023) 0.868	0.007 (-0.024, 0.038) 0.644
Tertile 3	-0.141 (-0.626, 0.345) 0.570	-0.224 (-0.725, 0.276) 0.380	-0.035 (-0.829, 0.759) 0.931	0.001 (-0.020, 0.023) 0.913	0.017 (-0.005, 0.039) 0.126	0.019 (-0.017, 0.056) 0.305
*p for trend*	0.555	0.357	0.883	0.889	0.114	0.281
*p for interaction*	0.019	0.032	0.031	0.003	0.073	0.036
**Subgroup analysis stratified by BMI *^d^***						
**Normal weight (BMI <25.0 kg/m²)**						
ln sPA	-1.376 (-2.173, -0.578) <0.001	-1.405 (-2.225, -0.584) <0.001	-1.209 (-2.517, 0.098) 0.072	0.046 (0.010, 0.081) 0.013	0.073 (0.038, 0.108) <0.001	0.080 (0.023, 0.136) 0.006
ln sPA categories						
Tertile 1	Reference	Reference	Reference	Reference	Reference	Reference
Tertile 2	-0.437 (-0.976, 0.103) 0.114	-0.449 (-1.011, 0.114) 0.119	-0.281 (-1.089, 0.527) 0.496	0.001 (-0.023, 0.026) 0.904	0.017 (-0.007, 0.042) 0.159	0.017 (-0.019, 0.052) 0.354
Tertile 3	-0.792 (-1.395, -0.189) 0.011	-0.768 (-1.393, -0.143) 0.017	-0.617 (-1.641, 0.408) 0.240	0.017 (-0.010, 0.044) 0.223	0.037 (0.010, 0.064) 0.007	0.036 (-0.009, 0.080) 0.122
*p for trend*	0.009	0.015	0.236	0.252	0.007	0.120
**Overweight (BMI 25.0–29.9 kg/m²)**						
ln sPA	-0.900 (-1.574, -0.226) 0.009	-0.943 (-1.623, -0.262) 0.007	-0.763 (-1.927, 0.402) 0.201	0.054 (0.021, 0.088) 0.002	0.060 (0.028, 0.092) <0.001	0.057 (0.002, 0.111) 0.045
ln sPA categories						
Tertile 1	Reference	Reference	Reference	Reference	Reference	Reference
Tertile 2	-0.294 (-0.863, 0.274) 0.311	-0.223 (-0.793, 0.348) 0.446	0.062 (-0.674, 0.799) 0.869	0.006 (-0.023, 0.034) 0.689	0.011 (-0.015, 0.038) 0.404	0.004 (-0.031, 0.039) 0.815
Tertile 3	-0.714 (-1.252, -0.176) 0.010	-0.688 (-1.225, -0.151) 0.013	-0.274 (-1.122, 0.575) 0.528	0.038 (0.011, 0.065) 0.006	0.040 (0.014, 0.065) 0.002	0.025 (-0.015, 0.065) 0.216
*p for trend*	0.010	0.012	0.497	0.006	0.002	0.199
**Obese (BMI >29.9 kg/m²)**						
ln sPA	-0.887 (-1.646, -0.129) 0.023	-1.034 (-1.803, -0.266) 0.009	-1.160 (-2.224, -0.097) 0.034	0.048 (0.015, 0.082) 0.005	0.059 (0.026, 0.093) <0.001	0.071 (0.024, 0.117) 0.003
ln sPA categories						
Tertile 1	Reference	Reference	Reference	Reference	Reference	Reference
Tertile 2	0.006 (-0.604, 0.616) 0.984	-0.153 (-0.761, 0.455) 0.622	-0.348 (-1.092, 0.397) 0.362	-0.001 (-0.028, 0.026) 0.931	0.002 (-0.024, 0.029) 0.864	0.013 (-0.020, 0.045) 0.451
Tertile 3	-0.650 (-1.251, -0.048) 0.035	-0.756 (-1.366, -0.146) 0.016	-1.014 (-1.880, -0.148) 0.023	0.027 (0.001, 0.054) 0.046	0.035 (0.008, 0.061) 0.012	0.046 (0.007, 0.084) 0.021
*p for trend*	0.020	0.010	0.018	0.027	0.006	0.015
*p for interaction*	0.601	0.719	0.641	0.936	0.844	0.832

a Model 1: no covariates were adjusted.

b Model 2: age, gender, and race/ethnicity were adjusted.

c Model 3: age, gender, race/ethnicity, education, marital status, poverty-to-income ratio, mean arterial pressure, body mass index, waist circumference, alcohol use, smoke, alanine aminotransferase, aspartate aminotransferase, total cholesterol, glucose, glycohemoglobin, creatinine, and urine iodin concentration were adjusted.

d There were 12 missing data of BMI.

FT3, free triiodothyronine; FT4, free thyroxine; sPA, serum palmitic acid; BMI, body mass index; CI, confidence interval.

## Discussion

The present retrospective investigation aimed to assess the relationship between sPA and thyroid function in a nationally representative sample of the population in the USA. In the 737 adult participants (age ≥18), we noted a significant association between sPA and FT4, and the FT3/FT4 ratio throughout multiple analyses. Subgroup analysis stratified by gender and BMI category indicated that the FT4 level and the FT3/FT4 ratio decreased and increased prominently in male and obese participants with higher sPA, respectively. Furthermore, interaction terms suggested that gender might influence the aforementioned associations.

In recent years, there has been an emerging focus on the effect of a high-fat diet (HFD) on human diseases, which could promote the disorders of lipid metabolism and triglyceride accumulation in non-adipose tissues (7, 35). In addition, increasing evidence has demonstrated the close relationship between lipid metabolism disorder and thyroid dysfunction, especially hypothyroidism or SCH (7, 35). In a cohort of 24,100 Chinese subjects with similar and stable iodine nutrition status, Zhao et al. (22) illustrated the significant positive association between the elevation of serum triglyceride levels and the risk for SCH. Similarly, Lai et al. (36) and Kota et al. (37) observed higher TSH levels in the participants with hypertriglyceridemia, which further suggested that the thyroid gland might be the victim of lipotoxicity. Primary thyroid hypofunction was also reported in diet-induced obese (DIO) mice, as well as the increased expression levels of the key molecules in the thyroid gland such as the TSH receptor, TPO, NIS, and Tg (38). However, in the experiments about the effect of short-term and long-term HFD feeding on obesity-prone (OP) and obesity-resistant (OR) mice, Xia et al. (39) illustrated suppressed NIS, TPO, and Tg mRNA levels in OP mice, and contradictory observations might be speculated to the upregulated TSH level (23). As shown in **Table 1**, this work also observed a slightly increased TSH level within the normal range in elevated ln sPA tertile; however, the statistical difference was insignificant (*p* = 0.081). On the other hand, the significant negative association between sPA and FT4 was considered to support the close correlation between lipotoxicity and hypothyroidism or SCH, and the hypothesis is desired in future studies.

As a representative SFA, PA has been widely applied in experiments investigating the underlying mechanisms of lipotoxicity on cellular damage (40). The present work showed that the elevated sPA level was significantly related to decreased FT4 (*p* < 0.001) and increased FT3/FT4 ratio (*p* = 0.001) after adjusting covariates, and the potential mechanism could be elucidated in some other studies. For instance, in human thyrocytes, Zhao et al. (23) demonstrated that PA could suppress NIS, TPO, and Tg mRNA levels, as well as promote intracellular lipid accumulation. Several studies have illustrated that endoplasmic reticulum (ER) stress could inhibit the expression of the three key molecules and their regulators in the thyrocytes (41, 42). In addition, ER stress was also observed in HFD rats and PA-treated thyrocytes, along with the increased degradation rate of Tg, which further indicated that the effect of ER stress on HFD-induced hypothyroidism might contribute to a decrease in the production of Tg (35).

As demonstrated in **Tables 1**, **3**, the subjects with elevated sPA level were more likely to have larger BMI and waist circumference, and the relationship between sPA and thyroid dysfunction was more significant in obese and male participants; several investigations might provide some implications to the underlying mechanism. As is well known, obesity results from an imbalance between caloric intake and caloric expenditure (43), and thyroid hormones (THs) play a pivotal role in modulating the energy balance, appetite, basal metabolic rate (BMR), and lipid metabolism (44). Changes in THs in obese subjects have been reported in various epidemiological studies, and Walczak et al. (44) further observe that obesity through lipotoxicity may lead to thyroid dysfunction and promote the development of SCH. Moreover, in the contralateral lobe of thyroid tissue derived from patients with PTMC, Lee et al. (38) observed the expansion of interfollicular adipose (IFA) depot and steatosis in thyroid follicular cells (thyroid steatosis, TS) in the majority of obese patients (BMI ≥25.0 kg/m²). Furthermore, SCH and morphological changes in the thyroid tissue were also documented in the male obese mice, which could not be remitted by peroxisome proliferator-activated receptor γ (PPARγ) agonist administration (38).

The findings of the present work should be interpreted cautiously because of some limitations. First, for the cross-sectional design of NHANES, the causal relationships between sPA and thyroid parameters could not be established. Hence, a cohort study with long observation periods and a larger sample size is desired in the future study. Second, the information on medical history, SES, marital status, tobacco consumption, and alcohol use was self-reported data obtained from questionnaires; thus, recall bias and several other errors could not be ruled out. Subsequently, we excluded children and pregnant participants on account of the uncertain influence on thyroid function, and further research is needed on these populations.

## Conclusion

In conclusion, we demonstrated the significant negative and positive relationship between sPA and FT4, and the FT3/FT4 ratio, respectively, in a representative population in the US. These results did not change on multiple imputations. In the subgroup analyses, the aforementioned associations were more remarkable in male and obese subjects. Our findings supported the close relationship between lipotoxicity and hypothyroidism or SCH, and further studies are needed to confirm this deduction. In addition, further studies are needed to verify the causal association between PA and thyroid function.

## Data Availability Statement

The original contributions presented in the study are included in the article/**Supplementary Material**. Further inquiries can be directed to the corresponding authors.

## Author Contributions

All authors listed have made a substantial, direct, and intellectual contribution to the work, and approved it for publication.

## Funding

This work was supported by grants from the Natural Science Foundation of Jiangsu Province (BK20181506) and Project of Jiangsu Province Hospital of Chinese Medicine (Y21024).

## Conflict of Interest

The authors declare that the research was conducted in the absence of any commercial or financial relationships that could be construed as a potential conflict of interest.

## Publisher’s Note

All claims expressed in this article are solely those of the authors and do not necessarily represent those of their affiliated organizations, or those of the publisher, the editors and the reviewers. Any product that may be evaluated in this article, or claim that may be made by its manufacturer, is not guaranteed or endorsed by the publisher.
