# Supplementary material for: The Association Between Serum Palmitic Acid and Thyroid Function
Source: Front Endocrinol (Lausanne). 2022 May 3;13:860634. doi: 10.3389/fendo.2022.860634 (PMC9110841; doi:10.3389/fendo.2022.860634)
Supplement: Supplementary file 1 [file Table_1.docx]

| Table S1 Baseline characteristics of study population in crude data and 5 models. | | | | | | | | |
| --- | --- | --- | --- | --- | --- | --- | --- | --- |
| Characteristics | Model | | | | | | | *p-value* |
|  | Crude | Ⅰ | Ⅱ | Ⅲ | Ⅳ | Ⅴ | Overall |  |
| N | 737 | 737 | 737 | 737 | 737 | 737 | 4422 |  |
| **Demographics** | | | | | | | | |
| Gender (%) |  |  |  |  |  |  |  | 1 |
| Male | 380 (51.56%)^a^ | 380 (51.56%) | 380 (51.56%) | 380 (51.56%) | 380 (51.56%) | 2280  (51.56%) | 2280  (51.56%) |  |
| Female | 357 (48.44%) | 357 (48.44%) | 357 (48.44%) | 357 (48.44%) | 357 (48.44%) | 2142 (48.44%) | 2142 (48.44%) |  |
| Age, years | 46 [30-63]^b^ | 46 [30-63] | 46 [30-63] | 46 [30-63] | 46 [30-63] | 46 [30-63] | 46 [30-63] | 1 |
| Race (%) |  |  |  |  |  |  |  | 1 |
| Non-Hispanic-White | 69  (9.36%) | 69  (9.36%) | 69  (9.36%) | 69  (9.36%) | 69  (9.36%) | 414 (9.36%) | 414 (9.36%) |  |
| Non-Hispanic Black | 70  (9.50%) | 70  (9.50%) | 70  (9.50%) | 70  (9.50%) | 70  (9.50%) | 420 (9.50%) | 420 (9.50%) |  |
| Mexican American | 302 (40.98%) | 302 (40.98%) | 302 (40.98%) | 302 (40.98%) | 302 (40.98%) | 1812 (40.98%) | 1812 (40.98%) |  |
| Other Hispanic | 171 (23.20%) | 171 (23.20%) | 171 (23.20%) | 171 (23.20%) | 171 (23.20%) | 1026 (23.20%) | 1026 (23.20%) |  |
| Other races | 125 (16.96%) | 125 (16.96%) | 125 (16.96%) | 125 (16.96%) | 125 (16.96%) | 750 (16.96%) | 750 (16.96%) |  |
| Education  (%) |  |  |  |  |  |  |  | 1 |
| <High school diploma | 172  (23.37%) | 172 (23.4%) | 172 (23.34%) | 172 (23.34%) | 172 (23.34%) | 1032 (23.34%) | 1032 (23.34%) |  |
| High school diploma | 146 (19.84%) | 147 (19.945%) | 147 (19.95%) | 146 (19.81%) | 146 (19.81%) | 879 (19.88%) | 879 (19.88%) |  |
| >High school diploma | 418 (56.79%) | 418 (56.72%) | 418 (56.72%) | 419 (56.85%) | 419 (56.85%) | 2510 (56.78%) | 2510 (56.77%) |  |
| Marital status (%) |  |  |  |  |  |  |  | 1 |
| Married | 353 (50.50%) | 367 (49.80%) | 371 (50.34%) | 373 (50.61%) | 371 (50.34%) | 2206 (50.32%) | 2206 (50.32%) |  |
| Unmarried | 346 (49.50%) | 370 (50.20%) | 366 (49.66%) | 364 (49.39%) | 366 (49.66%) | 2178 (49.68%) | 2178 (49.681%) |  |
| PIR (%) |  |  |  |  |  |  |  | 0.92 |
| 0-1 | 166 (24.67%) | 180 (24.42%) | 186 (25.24%) | 181 (24.56%) | 190 (25.78%) | 1087 (24.94%) | 1087 (24.94%) |  |
| ＞1 | 507 (75.33%) | 557 (75.58%) | 551 (74.76%) | 556 (75.44%) | 547 (74.22%) | 3271 (75.06%) | 3271 (75.06%) |  |
| Alcohol (%) |  |  |  |  |  |  |  | 0.97 |
| <12 drinks per year | 505 (74.82%) | 546 (74.08%) | 550 (74.63%) | 540 (73.27%) | 543 (73.68%) | 3223 (73.92%) | 3223 (73.92%) |  |
| ≥12 drinks per year | 170 (25.19%) | 191 (25.92%) | 187 (25.37%) | 197 (26.73%) | 194 (26.32%) | 1137 (26.08%) | 1137 (26.08%) |  |
| BMI, kg/m² | 27.40 [23.70-32.10] | 27.40 [23.70-32.10] | 27.40 [23.70-32.10] | 27.40 [23.70-32.10] | 27.40 [23.70-32.10] | 27.4 [23.70-32.10] | 27.4 [23.70-32.10] | 1 |
| WC, cm | 96.50 [86.20-107.60] | 96.50 [86.20-107.60] | 96.50 [86.20-107.60] | 96.50 [86.20-107.60] | 96.50 [86.20-107.60] | 96.50 [86.20-107.60] | 96.50 [86.20-107.60] | 1 |
| MAP, mmHg | 86.00 [79.11-93.78] | 86.00 [79.11-93.78] | 86.00 [79.11-93.78] | 86.00 [79.11-93.78] | 86.00 [79.11-93.78] | 86.00 [79.11-93.78] | 86.00 [79.11-93.78] | 1 |
| Smoke (%) |  |  |  |  |  |  |  | 1 |
| Never | 389 (55.57%) | 420 (56.99%) | 417 (56.58%) | 420 (56.99%) | 426 (57.80%) | 2498 (56.97%) | 2498 (56.97%) |  |
| Former | 163 (23.29%) | 165 (22.39%) | 167 (22.66%) | 167 (22.66%) | 163 (22.12%) | 988 (22.53%) | 988 (22.53%) |  |
| Current | 148 (21.14%) | 152 (20.62%) | 153 (20.76%) | 150 (20.35%) | 148 (20.08%) | 899 (20.50%) | 899 (20.50%) |  |
| Congestive Heart Failure (%) |  |  |  |  |  |  |  | 0.473 |
| No | 19  (2.72%) | 29  (3.93%) | 28  (3.78%) | 30  (4.07%) | 34  (4.61%) | 174 (3.97%) | 174 (3.97%) |  |
| Yes | 680 (97.28%) | 708 (96.07%) | 709 (96.20%) | 707 (95.93%) | 703 (95.39%) | 4210 (96.03%) | 4210 (96.03%) |  |
| Coronary Heart Disease (%) |  |  |  |  |  |  |  | 0.758 |
| No | 29  (4.16%) | 42  (5.70%) | 41  (5.56%) | 35  (4.75%) | 34  (4.61%) | 218 (4.98%) | 218 (4.98%) |  |
| Yes | 668 (95.84%) | 695 (94.30%) | 696 (94.44%) | 702 (95.25%) | 703 (95.39%) | 4164 (95.03%) | 4164 (95.03%) |  |
| Angina pectoris (%) |  |  |  |  |  |  |  | 0.103 |
| No | 16  (2.29%) | 45  (6.11%) | 37  (5.02%) | 39  (5.29%) | 30  (4.07%) | 198 (4.52%) | 198 (4.52%) |  |
| Yes | 684 (97.71%) | 692 (93.89%) | 700 (94.98%) | 698 (94.71%) | 707 (95.93%) | 4187 (95.49%) | 4187 (95.46%) |  |
| Heart Attack  (%) |  |  |  |  |  |  |  | 0.697 |
| No | 29  (4.14%) | 42  (5.70%) | 44  (5.97%) | 42  (5.70%) | 39  (5.30%) | 238 (5.43%) | 238 (5.43%) |  |
| Yes | 671 (95.86%) | 695 (94.30%) | 693 (94.03%) | 695 (94.30%) | 698 (94.71%) | 4147 (94.57%) | 4147 (94.57%) |  |
| Cancer (%) |  |  |  |  |  |  |  |  |
| No | 53  (7.57%) | 73  (9.91%) | 77 (10.45%) | 79 (10.72%) | 79 (10.72%) | 443 (10.10%) | 443 (10.10%) | 0.258 |
| Yes | 647 (92.43%) | 664 (90.10%) | 660 (89.55%) | 658 (89.28%) | 658 (89.28%) | 3942 (89.90%) | 3942 (89.90%) |  |
| **Laboratory indices** | | | | | | | | |
| CR, mg/dl | 0.84 [0.71-0.97] | 0.84 [0.71-0.97] | 0.84 [0.71-0.97] | 0.84 [0.71-0.97] | 0.84 [0.71-0.97] | 0.84 [0.71-0.97] | 0.84 [0.71-0.97] | 1 |
| Glucose, mg/dl | 93.00 [86.00-103.00] | 93.00 [86.00-103.00] | 93.00 [86.00-103.00] | 93.00 [86.00-103.00] | 93.00 [86.00-103.00] | 93.00 [86.00-103.00] | 93.00 [86.00-103.00] | 1 |
| Glycohemoglobin, % | 5.50 [5.20-6.00] | 5.50 [5.20-6.00] | 5.50 [5.20-6.00] | 5.50 [5.20-6.00] | 5.50 [5.20-6.00] | 5.50 [5.20-6.00] | 5.50 [5.20-6.00] | 1 |
| ALT, u/l | 20.00 [16.00-28.00] | 20.00 [16.00-28.00] | 20.00 [16.00-28.00] | 20.00 [16.00-28.00] | 20.00 [16.00-28.00] | 20.00 [16.00-28.00] | 20.00 [16.00-28.00] | 1 |
| AST, u/l | 23.00 [19.00-27.00] | 23.00 [19.00-27.00] | 23.00 [19.00-27.00] | 23.00 [19.00-27.00] | 23.00 [19.00-27.00] | 23.00 [19.00-27.00] | 23.00 [19.00-27.00] | 1 |
| UIC (%) |  |  |  |  |  |  |  | 1 |
| < 99 ug/L | 272 (37.31%) | 277 (37.59%) | 276 (37.45%) | 275 (37.31%) | 275 (37.31%) | 1652 (37.43%) | 1652 (37.43%) |  |
| 99-199 ug/L | 227 (31.14%) | 229 (31.07%) | 230 (31.21%) | 229 (31.07%) | 228 (30.94%) | 1371 (31.06%) | 1371 (31.06%) |  |
| >199 ug/L | 230 (31.55%) | 231 (31.34%) | 231 (31.34%) | 233 (31.62%) | 234 (31.75%) | 1391 (31.51%) | 1391 (31.51%) |  |
| TC, mg/dl | 189.00 [162.00-216.00] | 189.00 [162.00-216.00] | 189.00 [162.00-216.00] | 189.00 [162.00-216.00] | 189.00 [162.00-216.00] | 189.00 [162.00-216.00] | 189.00 [162.00-216.00] | 1 |

^a^ Actual frequency (weighted percentage).

^b^ Weighted median [95% CI for median].

Abbreviations: PIR, poverty-to-income ratio; BMI, body mass index; MAP, mean arterial pressure; CR, creatinine; ALT, alanine aminotransferase; AST, aspartate aminotransferase; TC, total serum cholesterol; UIC, urine iodin concentration.
