# Supplementary material for: The Association Between Serum Palmitic Acid and Thyroid Function
Source: Front Endocrinol (Lausanne). 2022 May 3;13:860634. doi: 10.3389/fendo.2022.860634 (PMC9110841; doi:10.3389/fendo.2022.860634)
Supplement: Supplementary file 2 [file Table_2.docx]

**Table S2 The association between ln sPA and FT4, FT3/FT4 in 5 models.**

|  | Model 1^a^  β (95% CI) *p-Value* | Model 2^b^  β (95% CI) *p-Value* | Model 3^c^  β (95% CI) *p-Value* |
| --- | --- | --- | --- |
| **FT4, pmol/L** | | | |
| Model Ⅰ |  |  |  |
| ln sPA | -1.041 (-1.465, -0.617) <0.001 | -1.090 (-1.516, -0.663) <0.001 | -0.983 (-1.542, -0.425) <0.001 |
| ln sPA categories |  |  |  |
| Tertile 1 | Reference | Reference | Reference |
| Tertile 2 | -0.234 (-0.561, 0.093) 0.162 | -0.218 (-0.544, 0.108) 0.190 | -0.141 (-0.494, 0.212) 0.433 |
| Tertile 3 | -0.751 (-1.080, -0.422) <0.001 | -0.726 (-1.057, -0.394) <0.001 | -0.603 (-1.034, -0.173) 0.006 |
| *p for trend* | <0.001 | <0.001 | 0.006 |
| Model Ⅱ |  |  |  |
| ln sPA | -1.041 (-1.465, -0.617) <0.001 | -1.090 (-1.516, -0.663) <0.001 | -1.015 (-1.575, -0.455) <0.001 |
| ln sPA categories |  |  |  |
| Tertile 1 | Reference | Reference | Reference |
| Tertile 2 | -0.234 (-0.561, 0.093) 0.162 | -0.218 (-0.544, 0.108) 0.190 | -0.151 (-0.506, 0.204) 0.404 |
| Tertile 3 | -0.751 (-1.080, -0.422) <0.001 | -0.726 (-1.057, -0.394) <0.001 | -0.618 (-1.050, -0.186) 0.005 |
| *p for trend* | <0.001 | <0.001 | 0.005 |
| Model Ⅲ |  |  |  |
| ln sPA | -1.041 (-1.465, -0.617) <0.001 | -1.090 (-1.516, -0.663) <0.001 | -0.990 (-1.549, -0.430) <0.001 |
| ln sPA categories |  |  |  |
| Tertile 1 | Reference | Reference | Reference |
| Tertile 2 | -0.234 (-0.561, 0.093) 0.162 | -0.218 (-0.544, 0.108) 0.190 | -0.142 (-0.497, 0.212) 0.432 |
| Tertile 3 | -0.751 (-1.080, -0.422) <0.001 | -0.726 (-1.057, -0.394) <0.001 | -0.600 (-1.033, -0.168) 0.007 |
| *p for trend* | <0.001 | <0.001 | 0.006 |
| Model Ⅳ |  |  |  |
| ln sPA | -1.041 (-1.465, -0.617) <0.001 | -1.090 (-1.516, -0.663) <0.001 | -0.992 (-1.551, -0.434) <0.001 |
| ln sPA categories |  |  |  |
| Tertile 1 | Reference | Reference | Reference |
| Tertile 2 | -0.234 (-0.561, 0.093) 0.162 | -0.218 (-0.544, 0.108) 0.190 | -0.143 (-0.496, 0.211) 0.429 |
| Tertile 3 | -0.751 (-1.080, -0.422) <0.001 | -0.726 (-1.057, -0.394) <0.001 | -0.605 (-1.036, -0.175) 0.006 |
| *p for trend* | <0.001 | <0.001 | 0.006 |
| Model Ⅴ |  |  |  |
| ln sPA | -1.041 (-1.465, -0.617) <0.001 | -1.090 (-1.516, -0.663) <0.001 | -0.999 (-1.559, -0.440) <0.001 |
| ln sPA categories |  |  |  |
| Tertile 1 | Reference | Reference | Reference |
| Tertile 2 | -0.234 (-0.561, 0.093) 0.162 | -0.218 (-0.544, 0.108) 0.190 | -0.145 (-0.500, 0.209) 0.422 |
| Tertile 3 | -0.751 (-1.080, -0.422) <0.001 | -0.726 (-1.057, -0.394) <0.001 | -0.608 (-1.040, -0.176) 0.006 |
| *p for trend* | <0.001 | <0.001 | 0.005 |
| **FT3/FT4** | | | |
| Model Ⅰ |  |  |  |
| ln sPA | 0.051 (0.032, 0.071) <0.001 | 0.064 (0.045, 0.083) <0.001 | 0.064 (0.040, 0.088) <0.001 |
| ln sPA categories |  |  |  |
| Tertile 1 | Reference | Reference | Reference |
| Tertile 2 | 0.002 (-0.013, 0.017) 0.772 | 0.011 (-0.004, 0.025) 0.146 | 0.008 (-0.008, 0.023) 0.338 |
| Tertile 3 | 0.030 (0.015, 0.045) <0.001 | 0.039 (0.024, 0.054) <0.001 | 0.033 (0.015, 0.052) <0.001 |
| *p for trend* | <0.001 | <0.001 | <0.001 |
| Model Ⅱ |  |  |  |
| ln sPA | 0.051 (0.032, 0.071) <0.001 | 0.064 (0.045, 0.083) <0.001 | 0.066 (0.041, 0.090) <0.001 |
| ln sPA categories |  |  |  |
| Tertile 1 | Reference | Reference | Reference |
| Tertile 2 | 0.002 (-0.013, 0.017) 0.772 | 0.011 (-0.004, 0.025) 0.146 | 0.008 (-0.007, 0.024) 0.299 |
| Tertile 3 | 0.030 (0.015, 0.045) <0.001 | 0.039 (0.024, 0.054) <0.001 | 0.035 (0.016, 0.054) <0.001 |
| *p for trend* | <0.001 | <0.001 | <0.001 |
| Model Ⅲ |  |  |  |
| ln sPA | 0.051 (0.032, 0.071) <0.001 | 0.064 (0.045, 0.083) <0.001 | 0.065 (0.041, 0.090) <0.001 |
| ln sPA categories |  |  |  |
| Tertile 1 | Reference | Reference | Reference |
| Tertile 2 | 0.002 (-0.013, 0.017) 0.772 | 0.011 (-0.004, 0.025) 0.146 | 0.008 (-0.008, 0.023) 0.331 |
| Tertile 3 | 0.030 (0.015, 0.045) <0.001 | 0.039 (0.024, 0.054) <0.001 | 0.034 (0.015, 0.053) <0.001 |
| *p for trend* | <0.001 | <0.001 | <0.001 |
| Model Ⅳ |  |  |  |
| ln sPA | 0.051 (0.032, 0.071) <0.001 | 0.064 (0.045, 0.083) <0.001 | 0.065 (0.041, 0.089) <0.001 |
| ln sPA categories |  |  |  |
| Tertile 1 | Reference | Reference | Reference |
| Tertile 2 | 0.002 (-0.013, 0.017) 0.772 | 0.011 (-0.004, 0.025) 0.146 | 0.008 (-0.008, 0.024) 0.318 |
| Tertile 3 | 0.030 (0.015, 0.045) <0.001 | 0.039 (0.024, 0.054) <0.001 | 0.034 (0.015, 0.053) <0.001 |
| *p for trend* | <0.001 | <0.001 | <0.001 |
| Model Ⅴ |  |  |  |
| ln sPA | 0.051 (0.032, 0.071) <0.001 | 0.064 (0.045, 0.083) <0.001 | 0.065 (0.041, 0.090) <0.001 |
| ln sPA categories |  |  |  |
| Tertile 1 | Reference | Reference | Reference |
| Tertile 2 | 0.002 (-0.013, 0.017) 0.772 | 0.011 (-0.004, 0.025) 0.146 | 0.008 (-0.008, 0.024) 0.310 |
| Tertile 3 | 0.030 (0.015, 0.045) <0.001 | 0.039 (0.024, 0.054) <0.001 | 0.034 (0.015, 0.053) <0.001 |
| *p for trend* | <0.001 | <0.001 | <0.001 |

^a^Model 1: no covariates were adjusted.

^b^Model 2: age, gender, and race/ethnicity were adjusted.

^c^Model 3: age, gender, race/ethnicity, education, marital status, poverty-to-income ratio, mean arterial pressure, body mass index, waist circumference, alcohol use, smoke, alanine aminotransferase, aspartate aminotransferase, total cholesterol, glucose, glycohemoglobin, creatinine, and urine iodin concentration were adjusted.

Abbreviations: FT3, free triiodothyronine; FT4, free thyroxine; sPA, serum palmitic acid.
