# Supplementary material for: The Association Between Serum Palmitic Acid and Thyroid Function
Source: Front Endocrinol (Lausanne). 2022 May 3;13:860634. doi: 10.3389/fendo.2022.860634 (PMC9110841; doi:10.3389/fendo.2022.860634)
Supplement: Supplementary file 3 [file Table_3.docx]

**Table S3 The association between ln sPA thyroid parameters except FT4 and FT3/FT4 ratio.**

| Exposure | Model 1^a^  β (95% CI) *p-Value* | Model 2^b^  β (95% CI) *p-Value* | Model 3^c^  β (95% CI) *p-Value* |  |
| --- | --- | --- | --- | --- |
| TSHI |  |  |  |  |
| ln sPA | -0.002 (-0.153, 0.150) 0.982 | -0.077 (-0.230, 0.077) 0.326 | -0.073 (-0.314, 0.168) 0.551 |  |
| ln sPA categories |  |  |  |  |
| Tertile 1 | Reference | Reference | Reference |  |
| Tertile 2 | 0.004 (-0.113, 0.121) 0.941 | -0.034 (-0.151, 0.083) 0.567 | -0.004 (-0.161, 0.152) 0.957 |  |
| Tertile 3 | -0.001 (-0.118, 0.117) 0.991 | -0.045 (-0.164, 0.074) 0.458 | -0.056 (-0.242, 0.130) 0.556 |  |
| TT4RI | | | |  |
| ln sPA | 0.146 (-5.193, 5.485) 0.957 | -1.913 (-7.384, 3.557) 0.493 | 2.072 (-7.195, 11.339) 0.661 |  |
| ln sPA categories |  |  |  |  |
| Tertile 1 | Reference | Reference | Reference |  |
| Tertile 2 | -0.239 (-4.353, 3.876) 0.910 | -1.549 (-5.709, 2.611) 0.466 | -0.525 (-6.537, 5.488) 0.864 |  |
| Tertile 3 | -0.310 (-4.453, 3.833) 0.884 | -1.758 (-5.991, 2.476) 0.416 | -0.599 (-7.767, 6.569) 0.870 |  |
| TFQI |  |  |  |  |
| ln sPA | -0.084 (-0.158, -0.009) 0.029 | -0.116 (-0.191, -0.041) 0.002 | -0.114 (-0.229, 0.001) 0.052 |  |
| ln sPA categories |  |  |  |  |
| Tertile 1 | Reference | Reference | Reference |  |
| Tertile 2 | -0.011 (-0.069, 0.047) 0.711 | -0.023 (-0.080, 0.034) 0.427 | -0.004 (-0.079, 0.071) 0.913 |  |
| Tertile 3 | -0.056 (-0.114, 0.002) 0.057 | -0.070 (-0.128, -0.012) 0.018 | -0.065 (-0.154, 0.024) 0.151 |  |
| FT3, pg/mL |  |  |  |  |
| ln sPA | 0.001 (-0.086, 0.089) 0.977 | 0.086 (0.009, 0.163) 0.030 | 0.129 (0.007, 0.252) 0.039 |  |
| ln sPA categories |  |  |  |  |
| Tertile 1 | Reference | Reference | Reference |  |
| Tertile 2 | -0.064 (-0.131, 0.003) 0.063 | 0.006 (-0.053, 0.065) 0.841 | 0.021 (-0.059, 0.101) 0.602 |  |
| Tertile 3 | -0.037 (-0.104, 0.031) 0.286 | 0.041 (-0.019, 0.101) 0.186 | 0.039 (-0.056, 0.134) 0.425 |  |
| Tg, ng/mL |  |  |  |  |
| ln sPA | 0.805 (-4.165, 5.775) 0.751 | 1.242 (-3.764, 6.248) 0.627 | -3.522 (-11.057, 4.012) 0.360 |  |
| ln sPA categories |  |  |  |  |
| Tertile 1 | Reference | Reference | Reference |  |
| Tertile 2 | 1.191 (-2.641, 5.024) 0.542 | 0.760 (-3.052, 4.572) 0.696 | 1.026 (-3.876, 5.927) 0.682 |  |
| Tertile 3 | 1.439 (-2.416, 5.294) 0.464 | 1.028 (-2.848, 4.903) 0.603 | -0.923 (-6.749, 4.904) 0.756 |  |
| TGAb, IU/mL |  |  |  |  |
| ln sPA | -3.489 (-20.501, 13.523) 0.688 | -7.987 (-25.553, 9.579) 0.373 | -3.384 (-33.376, 26.608) 0.825 |  |
| ln sPA categories |  |  |  |  |
| Tertile 1 | Reference | Reference | Reference |  |
| Tertile 2 | 6.504 (-6.597, 19.606) 0.331 | 4.339 (-9.022, 17.701) 0.525 | 3.823 (-15.632, 23.278) 0.700 |  |
| Tertile 3 | 0.432 (-12.759, 13.624) 0.949 | -2.153 (-15.749, 11.443) 0.756 | 2.388 (-20.805, 25.581) 0.840 |  |
| TSH, mIU/L |  |  |  |  |
| ln sPA | 0.221 (-0.250, 0.692) 0.358 | 0.033 (-0.450, 0.517) 0.892 | 0.414 (-0.404, 1.232) 0.322 |  |
| ln sPA categories |  |  |  |  |
| Tertile 1 | Reference | Reference | Reference |  |
| Tertile 2 | 0.042 (-0.321, 0.405) 0.820 | -0.084 (-0.451, 0.284) 0.656 | 0.022 (-0.509, 0.554) 0.934 |  |
| Tertile 3 | 0.125 (-0.240, 0.491) 0.502 | -0.016 (-0.390, 0.358) 0.931 | 0.122 (-0.512, 0.755) 0.707 |  |
| TPOAb, IU/mL |  |  |  |  |
| ln sPA | -0.543 (-24.803, 23.718) 0.965 | -2.531 (-27.590, 22.528) 0.843 | -13.611 (-53.636, 26.415) 0.505 | |
| ln sPA categories |  |  |  |  |
| Tertile 1 | Reference | Reference | Reference |  |
| Tertile 2 | 7.955 (-10.744, 26.655) 0.405 | 6.372 (-12.695, 25.438) 0.513 | -8.747 (-34.762, 17.268) 0.510 |  |
| Tertile 3 | 2.546 (-16.283, 21.375) 0.791 | 1.366 (-18.039, 20.772) 0.890 | -7.155 (-38.127, 23.816) 0.651 |  |
| TT3, ng/dL |  |  |  |  |
| ln sPA | 3.194 (-2.272, 8.659) 0.252 | 7.396 (2.043, 12.749) 0.007 | 10.659 (2.145, 19.173) 0.014 |  |
| ln sPA categories |  |  |  |  |
| Tertile 1 | Reference | Reference | Reference |  |
| Tertile 2 | -3.485 (-7.685, 0.714) 0.104 | -0.708 (-4.779, 3.363) 0.733 | 0.756 (-4.780, 6.291) 0.789 |  |
| Tertile 3 | 1.421 (-2.808, 5.650) 0.510 | 4.559 (0.416, 8.702) 0.031 | 5.462 (-1.137, 12.060) 0.105 |  |
| TT4, µg/dL |  |  |  |  |
| ln sPA | -0.192 (-0.554, 0.171) 0.300 | -0.191 (-0.565, 0.183) 0.318 | -0.102 (-0.678, 0.473) 0.728 |  |
| ln sPA categories |  |  |  |  |
| Tertile 1 | Reference | Reference | Reference |  |
| Tertile 2 | -0.185 (-0.464, 0.094) 0.194 | -0.201 (-0.486, 0.083) 0.165 | -0.177 (-0.550, 0.196) 0.353 |  |
| Tertile 3 | -0.089 (-0.370, 0.192) 0.535 | -0.100 (-0.389, 0.190) 0.500 | -0.017 (-0.462, 0.427) 0.939 |  |

^a^ Model 1: no covariates were adjusted.

^b^ Model 2: age, gender, and race/ethnicity were adjusted.

^c^ Model 3: age, gender, race/ethnicity, education, marital status, poverty-to-income ratio, mean arterial pressure, body mass index, waist circumference, alcohol use, smoke, alanine aminotransferase, aspartate aminotransferase, total cholesterol, glucose, glycohemoglobin, creatinine, and urine iodin concentration were adjusted.

Abbreviations: FT3, free triiodothyronine; FT4, free thyroxine; TSH, thyroid-stimulating hormone; TT3, Total T3; TT4, Total T4; Tg, thyroglobulin; TgAb, anti-thyroglobulin antibody; TPOAb, anti-thyroperoxidase antibody; TFQI, The thyroid feedback quantile-based index; TSHI, thyrotropin index; TT4RI, thyrotroph thyroxine resistance index; CI, confidence interval; sPA, serum palmitic acid.
